# Narrative review of recent developments and the future of penicillin allergy de-labelling by non-allergists

**DOI:** 10.1038/s44259-024-00035-6

**Published:** 2024-07-10

**Authors:** Neil Powell, Michael Blank, Akish Luintel, Shuayb Elkhalifa, Rashmeet Bhogal, Michael Wilcock, Michael Wakefield, Jonathan Sandoe

**Affiliations:** 1 Pharmacy Department, Royal Cornwall Hospital Trust, Truro, Cornwall UK; 2 Imperial College Healthcare NHS Trust, London, UK; 3 Institute of Global Health Innovation, Imperial College London, London, UK; 4 Centre for Musculoskeletal Research, Faculty of Biology, Medicine and Health, The University of Manchester, Manchester, UK; 5 Allergy and Immunology Department, Respiratory Institute, Cleveland Clinic Abu Dhabi, Abu Dhabi, United Arab Emirates; 6 The School of Pharmacy and Institute of Clinical Sciences, University of Birmingham, Birmingham, UK; 7 Respiratory Department, Harrogate and District NHS Foundation Trust, Harrogate, UK; 8 Healthcare associated infection group, Leeds institute of medical research, university of Leeds, Leeds, UK; 9 Department of Microbiology, Leeds Teaching Hospitals NHS Trust, Leeds, UK

**Keywords:** Antibiotics, Infection

## Abstract

This article outlines recent developments in non-allergist delivered penicillin allergy de-labelling (PADL), discusses remaining controversies and uncertainties and explores the future for non-allergist delivered PADL. Recent developments include national guidelines for non-allergist delivered PADL and validation of penicillin allergy risk assessment tools. Controversies remain on which penicillin allergy features are low risk of genuine allergy. In the future genetic or immunological tests may facilitate PADL.

## Introduction

Penicillin allergy (penA) records are common, with a reported prevalence in hospitalised patients between 3 and 16%^1^, but often incorrect with more than 90% able to tolerate penicillin after formal allergy testing^2^. PenA records are associated with patient, health-system and wider societal harms^3^. PenA testing traditionally includes blood tests, skin testing, and drug provocation testing, and delivered by allergists^4^. The paucity of allergists globally makes penA testing at any meaningful scale using traditional methods impractical^5^.

In 2008, Goldberg and Cohen demonstrated direct oral challenge (DOC) testing for penA without prior blood tests and skin testing, was a safe testing strategy in patients with a low-risk of serious reaction^6^. Thus, penA de-labelling (PADL) could be delivered to more patients and could be provided by non-allergist healthcare workers. A systematic review published in 2023 reported on the safety of non-allergist delivered PADL for 713 patients on history alone and 1288 via DOC^7^.

In this article we outline some of the recent developments in non-allergist delivered PADL, discuss any remaining controversies and uncertainties and explore the future for non-allergist delivered PADL.

## Recent developments

### Harms associated with penA labels and safety of non-allergist delivered PADL

In 2020 Krah et al. synthesised the published evidence on the influence of antibiotic allergy labels on antibiotic use and exposure, clinical outcomes, and healthcare-related costs^3^. Patients with antibiotic allergy labels received more broad-spectrum antibiotics, and there were associations between antibiotic allergy labels and increased length of hospitalisation, higher rates of intensive care unit (ICU) admission, higher hospital readmission rates, an increased risk of multidrug-resistant or opportunistic infections, and increased mortality^3^. Antibiotic allergy-labelled patients also incurred significantly higher drug costs^3^. A systematic review of non-allergist delivered PADL described 5019 patients de-labelled using a variety of methods; the adverse drug reaction rate was 1%, and none were serious^7^. Some of the PADL studies reported the positive impact PADL had on reducing broad spectrum antibiotic use and reducing antibiotic acquisition costs, but other impacts were not reported^7^.

### National non-allergist PADL guidelines

The evidence for the safety of non-allergy specialist delivered PADL has led to various national guidelines^8–13^, providing guidance on the required steps to de-label ‘low risk’ patients to enable non-allergy specialists to carry out this role safely. In the UK, the British Society for Allergy and Clinical Immunology guideline gives practical advice on how to set up a PADL service in hospitals^9^. The Scottish Antimicrobial Prescribing Group published a PADL guideline and included model letters for communicating test results to clinicians and patients^13^. The British Columbia PADL toolkit is for use by GPs and includes guidance on penA skin testing and useful information on setting up a service^14^. The Agency for Healthcare Research and Quality^15^ and the Dutch working party on antibiotic policy have provided materials for taking a comprehensive allergy history^16^. The American College of Allergy, Asthma, and Immunology (AAAAI) include PADL guidelines for adults and paediatric patients^17^. In contrast to the other guidelines, AAAAI provide guidance on the use of telemedicine for PADL which might prove useful for resource limited organisations, but need to be risk assessed prior to wider implementation^17^.

### Decision support tools

Recently, a number of validated decision support tools have been developed to help clinicians delabel patients using DOC (Table 1). PEN-FAST, the most studied, was initially retrospectively validated in adult outpatients; a score less than 3 had a negative predictive value (NPV) of 96.3%, however, only 4% of the test population had a history of anaphylaxis and only 9.3% had a positive penA test result^18^. The low prevalence of true allergy in the test population might make the high NPV less reliable. Some studies quote positive predictive values (PPV), since PPV falls with reduced prevalence of a condition (in this case, true penicillin allergy) the PPV can be used as an indication of the limitation of some validation studies. As these tools are used to rule out allergy rather than predict allergy, it is important that they are assessed using sufficient patients with a true allergy to ensure the NPV is accurate. A subsequent study used PEN-FAST in adults at higher risk of IgE mediated allergy (68.8% reported anaphylaxis) and had a reassuringly similar NPV^19^. PEN-FAST has been validated in other cohorts, including obstetrics^20^ and paediatrics^21^ (PEN-FAST was unreliable in paediatric populations under 12 years), and an ongoing trial in ICUs^22^. A randomised control trial (RCT) showed PEN-FAST to be non-inferior to penicillin skin testing; most had a score of ≤1, and patients with a history of anaphylaxis, severe delayed allergic reactions/ intolerances were excluded^23^. There are concerns that the decision support tools may miss delayed hypersensitivity reactions^24^. It is important that non-allergists have adequate training to be able to safely use these decision support tools. Owens et al. randomised healthcare workers, who did not have prior training in penicillin allergy de-labelling or use of the tool, to assess penA histories and assign appropriate management of eight clinical vignettes either using a decision support tool or without the tool^25^. The decision support tool reduced the number of major errors assigning the correct de-label method, but there was still a significant risk of HCWs opting for DDL or DOC in high-risk patients where the most appropriate management should have been outpatient allergist review^25^.

**Table 1:** Studies comparing penicillin allergy history with either drug challenge or skin testing

Author	Year	Tool validated	Cohort	Country	Tool’s NPV for penicillin allergy	Tool’s PPV for penicillin allergy
Trubiano et al.^75^	2020	PEN-FAST	Outpatient and inpatient	Australia and USA	96.3%	25.3%
Piotin et al.^19^	2021	PEN-FAST	Outpatient and inpatient	France	93%	81%
Copaescu et al.^21^	2022	PEN-FAST	Paediatrics	Canada	95%	5.87%
Mak et al.^20^	2022	PEN-FAST	Obstetrics	Canada	100%	Not stated
Su et al.^92^	2023	PEN-FAST	Outpatient (adults)	USA	100%	12.5%
Soria et al.^93^	2017	Locally developed tool	Outpatient (adults)	France	96.3%	21.3%
Siew et al.^94^	2019	Locally developed tool	Outpatient (adults)	UK	98.4%	Not stated
Mohamed et al.^95^	2019	Locally developed tool	Outpatient (adults)	UK	94%	Not stated
Stone et al.^96^	2020	Locally developed tool	ICU	USA	99%	Not stated
Stevenson et al.^97^	2020	Locally developed tool	Outpatient (adults)	Australia	94.7%	Not stated
Rosman et al.^98^	2021	Locally developed tool	Outpatient (adults)	Israel	95%	23%
Elkhalifa et al.^99^	2021	Drug-allergy app	Outpatient (adults)	UK	100%	79.7%

*NPV* Negative Predictive Value, *PPV* Positive Predictive Value, *ICU* Intensive Care Unit.

### UK PADL studies

The UK National Institute for Health and Care Research (NIHR) is funding research into PADL in the UK’s National Health Service, across a range of healthcare delivery settings: hospital inpatient care, outpatient services and primary care^26–28^. A Multicentre Study to Investigate Feasibility of a Protocol-Driven Multidisciplinary Service Model to Tackle ‘Spurious Penicillin Allergy’ in Secondary Care (SPACE study) looked at research nurses and research pharmacists delivering PADL in different secondary care settings; acute medical unit, surgical pre-op and haematology units^29^. The Allergy AntiBiotics And Microbial resistAnce (ALABAMA) programme is looking at the safety and effectiveness of a pre-emptive, penA assessment pathway initiated in primary care in advance of need, using an open label RCT^30^. The ALABAMA programme has taken an in-depth approach to understanding the barriers and facilitators to penA assessment both from a patient and clinician perspective, and to understand factors affecting antibiotic use after a negative test^31,32^. Removing Erroneous Penicillin Allergy Labels (REPeAL) is aiming to design, develop and implement an intervention that embeds PADL as a standard of care delivered by ward-based healthcare workers, also using a behavioural approach to intervention design^33^. When published, the results of these studies will add to the growing number of non-allergist PADL studies from around the world^7^.

### Education and training for non-allergists

National and Global standards for education and training for non-allergy specialist led PADL are not available. Heterogeneity in training needs for the different non-allergy specialists needs to be considered. Staicu et al., reviewed online PADL education resources for non-allergy specialists^34^. A penA toolkit and educational video provide insights into penA history taking, risk stratification, management of patients with a high-risk penA history, de-labelling protocols for patients with a low risk penA history, model letters for communication of the penA test outcome and raising awareness of false penicillin allergy labels amongst patients^34^. The British Society for Antimicrobial Chemotherapy recently launched a course aimed at non-allergy specialist led PADL and intended as a national platform to standardise education and training for non-allergy specialists^35^.

## Controversies

### Limitations and challenges in the diagnosis of penicillin allergy

There are several diagnostic challenges associated with penA diagnosis. Although detection of penicillin-specific IgE antibodies can be helpful in patients with genuine penA, low sensitivity and low specificity limit it’s utility to specialist settings^36^. Integrated methods, combining clinical history, laboratory testing, and drug provocation tests, are advocated for accurate penA diagnosis in high-risk patients, but there are variations in sensitivities, specificities, safety profiles, and patient outcomes between serum IgE testing and skin testing^37^. In low-risk patients, the predictive value of skin testing diminishes, and it is not uncommon to have positive serum penicillin specific IgE tests in a patient with a history not suggestive of an IgE-mediated allergy^6,38,39^. This discrepancy raises questions about the predictive value of these tests in patients with low-risk penA histories and complicate clinical decision-making, with drug provocation tests (DPTs) the gold standard test to rule out penA^40^.

### Penicillin allergy testing in low-risk patients: a call for a consensus

There is broad agreement on the need to risk stratify patients with a penA label to ensure only those with a very low risk of a serious reaction to penicillin are tested outside specialist settings. There is also agreement that such patients can receive a direct DPT without the need for prior skin testing and serum penicillin specific IgE. Beyond these broad principles there is heterogeneity in guidance and practice that hampers implementation and assessment of the effectiveness of interventions^41–43^. The differences reflect variation in the definitions used and in the general approach to risk; some approaches reflecting highly risk averse practice while others are more pragmatic. Studies differ in their inclusion of patients who report urticarial rashes, their definitions of ‘benign’ rashes, and the time period since reactions occurred that constitutes a low risk^42^. Most, but surprisingly not all, exclude patients with a history consistent with anaphylaxis^42^. Exclusion criteria for oral challenge testing based on comorbidities and current medication vary widely. To assess safety and effectiveness of penA assessment pathways more robustly, it would seem sensible to define a standard - against which future developments could be evaluated. There are now several published national and international PADL guidelines with consensus statements for some of the key elements of penicillin allergy de-labelling informed by recent research data and expert opinion. These might be improved further by undertaking a Delphi consensus or similar process to reach consensus on current issues where there remain inconsistencies between guidance and develop international guidelines^9,13,44–46^.

There is variability in DPT dosing and duration^41,43^. The BSACI guidance recommends either a single full oral dose, or graded oral dose, e.g. 10% of a full dose escalated at time intervals to a full single dose, based on local preference, with little evidence that one is safer than the other^9^. There is a lack of standardisation for duration of oral DPT which ranges from a single dose to five days^41^. A study by Fransson et al. demonstrated 45% of reactions following DPT occurred more than 3 days after the first dose, and argue these reactions might be missed without prolonged provocation^47^. If prolonged provocation is more sensitive in detecting delayed hypersensitivity this needs to be balanced against its potential negative impact on the microbiome, the costs of testing, and follow-up.

Duration of observation periods following DOC also vary; between 30 and 120 min, with most observing for 60 min post challenge^41^. Studies using 90 or 120 min have used allergy centre skin testing protocols, perhaps warranted in higher risk patient groups, but the rationale for these longer observation periods in these low-risk cohorts is not provided^48–52^. Two studies adopted a 30 min observation period: Mustafa et al.’s outpatient allergist delivered study argues the shorter 30 min observation period is beneficial for both patient convenience and patient flow, and none of their 185 patients given drug provocation tests had a reaction^38^. Brayson et al.’s pharmacist delivered inpatient study also adopted 30 min of direct observation with 30 min of self-monitoring^53^. None of the non-allergist delivered DOC studies testing low-risk patients have identified severe immediate reactions and have all have been carried out in hospitals with access to emergency assistance. Given the safety of DOC in low-risk patients and the environment that PADL is delivered, it may be possible to reduce observation periods to improve efficiency of PADL, but this would need prospective assessment in larger populations.

### Safe PADL environments

The requirements of a safe testing environment for patients with low risk penA assessment requires standardisation. While some guidelines recommend the availability of on-site resuscitation and critical care when delabelling^9^, others argue for a controlled environment with access to relevant medications (including steroids and adrenaline) and healthcare personnel^54^, and it remains controversial whether on-site critical care services are required for DPT in low-risk patients. Primary care and other community health services remain a mostly untapped area for delabelling, and consensus on the necessity of resuscitation resources is important. Other possible environments include nursing homes and pharmacies, where medications can be dispensed.

What oversight looks like remains controversial. The BSACI guideline for setting up PADL services in the UK by non-allergy specialists recommends working collaboratively with specialist allergy immunology services, which remains a challenge given the paucity of allergists^5,9^. Due to the heterogeneity in PADL protocols and unforeseen patient scenarios, there will be instances where patients with a penA label fall outside of the testing protocol for which it will become important to seek advice from allergy services but national strategic planning and the necessary governance for non-allergy specialist and allergy specialist PADL responsibilities will need to be in place to avoid over-burdening a service that is already over-stretched^41,42^.

### Legal implications of PADL

While hospitals and clinicians may be liable when harm is done giving a penicillin to a penA patient^55,56^, it is worth considering the converse: might a clinician be doing harm to a patient by *not* giving a penicillin to a patient who is mislabelled as penA and could be delabelled? A penA label that precludes access to a beta-lactam antibiotic could cause patient harm through necessitating the use of a less effective antibiotic or increase their risk of future AMR infections through unnecessary exposure to broader spectrum antibiotics. Increasing antimicrobial resistance means fewer available antibiotics, an incorrect penA might deny a patient the life-saving penicillin they need; these are unnecessary harms when the patient could be delabelled^57^. It is also worth considering the possibility that delabelling could go wrong, resulting in anaphylaxis or even death upon subsequent exposure to penicillin; it is precisely because of this possibility that informed consent, in which we balance harms against benefits, remains essential. In the vast majority of patients, the benefit of delabelling far outweighs the harms; maintenance of the status quo reminds one of the so-called ‘trolley-car’ problem in which a passive approach may not be the best option^57,58^.

## Future

### Empowering patients and HCWs

To optimise the impact of PADL, the irrational fear that penA labels generate in some HCWs needs to be overcome. Anaesthetists have reported unwillingness to use penicillin in patients who have been delabelled^59^. Perception of risk is a significant barrier to PADL^60–62^, even though the risk of harm from the penA label outweighs the risk of anaphylaxis during treatment in those who have been delabelled^40^. Better communication of the risks associated with delabelling will be needed to support clinicians who remain anxious about prescribing penicillins in this situation. Standardised processes of penA assessment need to be endorsed by policy makers and normalised in clinical practice to ensure optimal management of infections. Likewise, there are some patients who are unwilling to engage with PADL and who decline to take penicillin even if they have been delabelled; the numbers of such patients are small and may decline as PADL becomes normalised in clinical care^62^.

There is a growing body of literature demonstrating that patient empowerment in disease management has it’s a positive impact on care^63^. It may be possible to develop risk stratification tools that patients are able to use to risk stratify themselves and to de-label themselves on history alone, without DPT. Such an approach may be limited by a lack of confidence in the process, given the reluctance amongst some prescribers to prescribe penicillin even to patients with a negative DPTs, and potential for patients to continue to avoid penicillin without a DPT in a controlled environment^59,64^.

### In vitro diagnostics

Advancement in our knowledge of the genetic factors influencing hypersensitivity reactions may improve penA diagnosis and provide greater confidence with PADL^65,66^. HLA class I alleles, expressed on all nucleated cells, play a crucial role in adaptive immune responses. However, despite the increasing evidence of HLA involvement in drug-induced hypersensitivity, mechanistic details, especially regarding how genetic variation predisposes to specific drug reactions, remain unclear^67^. A robust association has been identified between self-reported penicillin allergy and an allele of the MHC class I gene HLA-B^66^. This association raises the possibility of a T cell-mediated process leading to delayed penicillin reactions, possibly triggered by an HLA-B∗55:01 restricted immune response to a prevalent pathogen earlier in life. Genetic correlations with autoimmune diseases like rheumatoid arthritis and psoriasis suggest underlying autoimmune factors in penA development^66^. These findings contribute to understanding the genetic architecture of penicillin allergy, urging further studies to elucidate the underlying immune processes and their evolution over time.

Basophil Activation Testing (BAT) has emerged as a valuable tool in the diagnosis of penA, offering insights into the immune response at the cellular level. In the context of penA, BAT offers advantages over traditional diagnostic methods, providing real-time information about the patient’s sensitivity to penicillin^68^.

Lymphocyte Transformation Testing (LTT) is a tool for assessing delayed hypersensitivity to penicillin, providing insights into the cellular immune response associated with hypersensitivity reactions. While LTT has shown promise, its clinical utility has not been established, with clinical validation required to establish the efficacy of LTT in penA diagnosis^69^. The development of more comprehensive in vitro tests that can simultaneously assess multiple pathways of hypersensitivity reactions represents a promising area of advancement^67^.

### Harnessing IT systems to improve penA documentation and PADL

The quality of allergy data on electronic health record systems (EHRs) is often poor and incomplete^70^. Improving the user interface within EHRs and provision of better healthcare worker training, may improve allergy documentation^71^. Another method, to improve data quality, is to employ artificial intelligence (AI) techniques such as natural language processing (NLP). Natural language processing is a branch of AI which employs various techniques to interrogate, extract and unlock meaning from free text^72^. There are many parts of an allergy history which may not be in structured sections and by harnessing NLP we may be able to enrich data quality. Goss et al. used NLP to extract and encode allergy information from clinical notes^73^. Relating to penA, Inglis et al. showed a NLP programme which accurately classified ADRs into intolerance and allergy with a 0.99 sensitivity and 0.96 specificity^74^. Employing NLP, in this manner could further enhance data quality, potentially refining alerting systems and recording. This may ensure that only pertinent patients trigger notifications not to prescribe penicillin, and the distinction between intolerance and allergy is highlighted to the clinician. This more accurate penA history data could then be used to flag those patients likely to be successfully de-labelled, with the incorporation of clinical decision support systems or risk scores, such as PENFAST^75^, further streamlining and expediting the PADL process. AI has been used to risk stratify small cohorts of penA patients^76–78^, but further research into the role of AI in risk stratification of penA patients, de-labelling false labels and communication of the outcome of the intervention is needed.

## Knowledge gaps

### Which HCW or group is best positioned to deliver PADL?

A range of healthcare workers have been involved in PADL: pharmacists, doctors, nurses, nurse practitioners, physician associates, medical students, and pharmacy students^7^. Approximately half the reported studies use multidisciplinary teams to deliver PADL and half unidisciplinary^7^. Multidisciplinary interventions involved at least one doctor, the majority of unidisciplinary studies were delivered by pharmacists, with all studies delivered in developed countries^7^. Two qualitative studies from the UK and US interviewed hospital doctors and pharmacists to understand the barriers and enablers to PADL. In both studies, doctors and pharmacists reported that PADL aligns with their role and that with adequate support and training they would deliver PADL^60^. In both studies doctors and pharmacists responded that they believed PADL required a collaborative approach^60,79^. Another US study interviewed doctors, nurses and pharmacists and used their findings to propose two interventions that would facilitate PADL of which one was to assign pharmacists and pharmacy technicians responsibility for PADL, recognising pharmacists as best placed to deliver PADL^61^. Ngassa et al. also recognised the role of the pharmacist in delivering PADL^62^. The majority of current evidence looks to support pharmacist-delivered PADL with support from doctors.

### The impact of penA labels in less developed countries is largely unknown

The prevalence of penicillin allergy labels and impact on antimicrobial stewardship is known for high income countries (HICs), but not for low- and middle-income countries (LMICs); nonetheless data from HICs have been extrapolated to LMICs, resulting in recommendations to implement PADL interventions^80^. The prevalence of PenA labels is not known in LMICs due to multiple challenges collecting the data^80,81^. Research into the prevalence of false PenA labels in LMICs is needed to plan PADL interventions.

### Ethnicity and diversity in PADL studies

PADL studies delivered by non-allergy specialists have been predominantly carried out in HIC’s including USA, Australia and UK and the study populations predominantly white or Caucasian, or ethnicity unreported (Table 2)^7^. There is a lack of representation from black, Asian and minority ethnic (BAME) populations and therefore the safety of PADL interventions cannot be extrapolated to these populations. The USA, UK and Australia make up approximately 5% of the World’s population (approximately 8 billion) with India and China making up approximately 25% of the world’s population collectively^82^. More recently, the first non-allergist delivered PADL study in Chinese patients reported the successful de-label of 144 low risk patients in Hong Hong^83^. Further research and validation of PADL interventions is needed in BAME populations to reliably ascertain the safety of these interventions and the benefits of de-labelling a false penA label.

**Table 2:** Studies that looked at penicillin allergy de-labelling with a direct oral penicillin challenge or validation of a penicillin allergy de-labelling toolkit

Study	Country	Total number of recruited patients	Ethnicity of recruited patients
Chua et al.^100^	Australia	1225	White - 1125 (91.8%)
Tucker et al.^101^	USA	402	Not reported
Mustafa et al.^38^	USA	363	Not reported
Sneddon et al.^13^	UK	112	Not reported
Brayson et al.^53^	UK	304	Not reported
Hearsey et al.^102^	UK	285	Not reported
Iammatteo et al.^103^	USA	155	Latino 58 (37%) White 43 (28%) Black 41 (26%) Multiracial 4 (3%) Unknown 9 (6%)
Kuruvilla et al.^104^	USA	50	Not reported
Trubiano et al.^75^	Australia	622	In the ‘univariate and multivariable analysis of features associated with a positive penicillin allergy test result in derivation and validation cohort’: Non-white race 29 (4.7%)
Savic et al.^105^	UK	74	Not reported.
Li et al.^50^	Australia	71	Not reported
du Plessis et al.^106^	New Zealand	250	Caucasian 116 (46%) Māori 37 (15%) Pacific Island 43 (17%) Asian 48 (19%) other 6 (2%)
Devchand et al.^107^	Australia	106	Not reported

### Feasibility of implementing a universal PADL toolkit across countries

The feasibility of implementing standardised PADL universally, especially in less developed countries, poses unique challenges and opportunities. PADL toolkits are designed to aid healthcare providers to deliver PADL and often include guidelines, diagnostic algorithms, patient questionnaires, and educational materials and have been shown to significantly reduce inappropriate penA labelling, leading to better antibiotic stewardship and patient outcomes^3,7^. Differences in healthcare systems, patient demographics, cultural beliefs, healthcare provider training and practices can affect the adaptability and effectiveness of PADL toolkits^8^. A universal PADL toolkit needs to be adaptable to various healthcare settings, resource levels, and cultural contexts. It should incorporate flexible guidelines and diagnostic criteria that can be tailored to local contexts. Successful implementation requires integration with existing healthcare practices and policies in different countries, and engagement with local healthcare professionals for customisation.

Conducting research and pilot studies in various countries can provide valuable insights into the toolkit’s effectiveness and areas for improvement, informing future adaptations^40^. The development and implementation of a universal PADL toolkit hold promise for improving global antibiotic stewardship and patient care. However, its success depends on adaptability to diverse healthcare environments, collaborative international efforts, and ongoing research and refinement.

### Does PADL improve patient outcomes?

The majority of studies assessing the impact of penA labels on health outcomes have been uncontrolled retrospective observational studies that can only reliably describe associations between penA status and adverse outcomes. Even when there has been careful consideration of potential confounding during analysis there remains the possibility of unmeasured cofounders^84,85^. One way to overcome the problem of ‘confounding by indication’ i.e. why the antibiotic was prescribed in the first place, is to study patients with a single clinical condition. When patients with community acquired pneumonia were studied, hospital admission and ICU admission were significantly greater in patients with a penA label^86^. If these observational studies are correct about the harms associated with penA labels, we do not yet know if the risk of worse health outcomes like mortality, hospital admission, MRSA infection, *C. difficile* infection is reduced by correcting penA status. The ALABAMA trial is a RCT of a penA assessment pathway versus usual clinical care and has several health outcomes as secondary outcome measures but it may be underpowered to detect an impact on these having been affected by the COVID-19 pandemic^87^. Larger RCTs may be needed to confirm the impact of delabelling on health outcomes and will certainly be needed to determine an effect on AMR, given the relatively small number of patients affected. The International Network of Antimicrobial Allergy Network study is an international case match study that aims to determine whether PADL improves patient outcomes^88^.

### The rising challenge of multiple antibiotic allergies

Patients with labels of penicillin and other antibiotic allergy are an increasing problem that can limit therapeutic options, especially in critical care and surgical prophylaxis; they also increase healthcare costs due to the use of broader-spectrum/ more expensive antibiotics^89^. Addressing the issue of multiple antibiotic allergies requires study of the mechanisms (including immunological) behind these allergies as well as diagnostic and management strategies^90,91^.

Diagnosing allergies to multiple antibiotics is complex. Conventional diagnostic methods, such as skin testing and specific IgE measurements, may not be sufficiently sensitive or specific, particularly for delayed-type hypersensitivity reactions. Structured patient interviews, skin testing, and DPTs are effective strategies for de-labelling. These methods have been successful in confirming the absence of true allergies in many patients previously labelled as multi-drug allergic^8^. DPTs are underutilized but crucial in the definitive diagnosis of drug allergies. However, concerns about patient safety, especially in cases with a history of severe reactions, limit their widespread use^40^.

Personalised medicine, including genetic testing and detailed immunological profiling, may offer new avenues for diagnosing and managing suspected true multiple antibiotic allergies. This approach could lead to more targeted and effective allergy assessments. Enhanced diagnostic accuracy, effective de-labelling strategies, and continued research are essential for better management of these patients and for improving overall antibiotic stewardship^90,91^.

## Discussion

The negative impact of penA labels on antimicrobial stewardship and patient safety are now well described in the literature. However, these data are from HICs with representation of minority ethic groups either very low or not reported and as such there is a gap in our understanding of the harms of penA and how to optimise PADL in these populations. Several countries have developed national guidelines, and researchers have validated decision support tools, that support the wider healthcare workforce to safely remove incorrect penA labels enabling more patients to receive first line penicillin antibiotics. Although studies have shown PADL increases the use of penicillin antibiotics it remains unknown whether this reverses the associated harms of penA labels. How best to train HCWS to deliver PADL, and the optimal testing strategy that enables the greatest number of patients to be de-labelled safely, are yet to be determined. DPT is the current gold standard for testing penA but there may be opportunity in the future to utilise genetic or immunological tests to facilitate PADL. Legal levers, patient empowerment and AI all offer opportunity to more widely disseminate PADL and there needs to be some thought given to how we tackle the problem of multiple antibiotic allergy labels.
